# Three new species of eriophyoid mites (Acari, Eriophyoidea) from Xinjiang Uygur Autonomous Region, China

**DOI:** 10.3897/zookeys.508.8940

**Published:** 2015-06-17

**Authors:** Ji-Wei Li, Zhen-Hui Wang, Xiao-Feng Xue, Jian-Ping Zhang

**Affiliations:** 1 Department of Plant Protection, College of Agriculture, Shihezi University, Shihezi, Xinjiang 832000, China; 2 Department of Entomology, Nanjing Agricultural University, Nanjing, Jiangsu 210095, China

**Keywords:** Taxonomy, Colomerini, Phyllocoptini, Rhyncaphytoptinae, Rosaceae

## Abstract

Three new species of eriophyoid mites from Xinjiang Uygur Autonomous Region, China, are described and illustrated. They are *Paracolomerus gonglius* **sp. n.** and *Phyllocoptruta beggerianae* **sp. n.** collected on *Rosa beggeriana* Schrenk ex Fisch. & C. A. Mey. (Rosaceae), and *Rhyncaphytoptus fuyuniensis* **sp. n.** collected on *Cotoneaster ignavus* E. L. Wolf (Rosaceae). All eriophyoid mites described here are vagrants on the undersurface of leaves and any apparent damage was not observed.

## Introduction

Eriophyoid mites (Acari: Eriophyoidea) have been recognized as important pests in agriculture and forestry all over the world (Lindquist et al. 1996). Their stylets are involved in piercing plant cells, injecting saliva into them and sucking cell sap (de Lillo et al. 2002). The saliva causes cytological, biochemical and physiological changes in the pierced plants (de Lillo and Monfreda 2004, Petanović and Kielkiewicz 2010a). Eriophyoid mites induce plant malformations as galls, complex symptoms or vector pathogens disturbing the normal growth of plants (Petanović and Kielkiewicz 2010b). This is the case of *Colomerus vitis* (Pagenstecher), *Aceria pallida* Keifer and *Tegolophus zizyphagus* (Keifer) which induce erinea, galls or leaf edge curls and cause economic losses to grape, matrimony vine and jujube, respectively, in Xinjiang (Lu and Mao 1990, Zang 1998, Yang et al. 2012). However, about half eriophyoid mite species are vagrants on the surface of leaves and do not cause any apparent damage (Huang 2008, Skoracka et al. 2010, Petanović and Kielkiewicz 2010b). These mites, occurring in a large amount, may cause non-distortive changes and affect the normal growth of the plants (Oldfield 1996). Usually eriophyoids are tiny in size and hard to see with unaided eyes. Sometimes their symptoms can be confused with those due to viruses, nutrient deficiency and physiological disorders (Van Leeuwen et al. 2010). Therefore, it is necessary to study the systematic account of Eriophyoidea for having a further contribute in better understanding their significance in Agriculture.

Kuang (1995) first explored and reported the eriophyoid mite fauna in Xinjiang. After that, a number of field surveys were conducted in the same area and further 31 species were reported so far. Out of 31 species, 1 species belongs to the family Phytoptidae, 2 species belong to the family Diptilomiopidae and 28 species belong to the family Eriophyidae (Table 1). The fact that more than 1000 species have been recognized from China (personal data of X.-F. Xue) suggests that many more areas need to be explored more carefully. For this purpose eriophyoid mites were collected by Ji-Wei Li from Tianshan Mountains, Altai Mountains, Farmlands and Gurbantunggut Desert of Xinjinag in 2013 and 2014.

**Figure 1.**
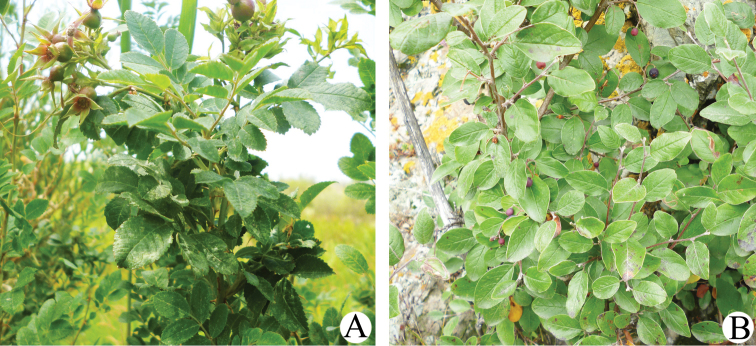
**A** *Rosa beggeriana* Schrenk ex Fisch. & C.A. Mey **B** *Cotoneaster ignavus* E.L. Wolf.

In the present study, we describe 2 new species of the genera *Paracolomerus* and *Phyllocoptruta* collected on *Rosa beggeriana* (Fig. 1) and one new species of the genus *Rhyncaphytoptus* collected on *Cotoneaster ignavus* (Fig. 1), all from Xinjiang. Also, this is the first description of the genus *Paracolomerus* from Rosaceae.

**Table 1.** List of eriophyoid mites from Xinjiang Uygur Autonomous Region.

Family	Subfamily	Tribe	Species	Reference
Phytoptidae	Nalepellinae	Nalepellini	*Setoptus koraiensis* Kuang & Hong, 1995	Xue et al. 2012a: 12.
Eriophyidae	Cecidophyinae	Colomerini	*Colomerus vitis* (Pagenstecher, 1857)	Kuang 1995: 42–43.
			*Paracolomerus gonglius* sp. n.	This study
	Eriophyinae	Eriophyini	*Eriophyes catacardiae* Keifer, 1962	Kuang 1995: 47.
		Aceriini	*Aceria bromi* Kuang & Zhang, 1992	Kuang 1995: 53–54.
			*Aceria changjiensis* Kuang & Pang, 1997	Kuang and Pang 1997: 230–231.
			*Aceria dispar* (Nalepa, 1891)	Song et al. 2008: 13.
			*Aceria haloxylonis* Xue, Zhang, Li & Hong, 2012	Xue et al. 2012b: 203–208.
			*Aceria nimia* Hall, 1967	Kuang et al. 2005: 29.
			*Aceria pallida* Keifer, 1964	Hong et al. 2006: 230.
			*Aceria tamaricis* (Trotter, 1901)	Kuang et al. 2005: 33–34.
			*Aceria tosichella* Keifer, 1969	Kuang 1995: 64–65.
	Phyllocoptinae	Phyllocoptini	*Calepitrimerus alchemillae* (Liro, 1940)	Kuang et al. 2005: 58–59.
			*Epitrimerus sabinae* Xue & Hong, 2005	Xue et al. 2012a: 31.
			*Phyllocoptes pyrivagrans* Kadono, 1985	Kuang et al. 2005: 68–69.
			*Phyllocoptruta beggerianae* sp. n.	This study
			*Proiectus tabulaeformis* Xue, Song, Amrine & Hong, 2007	Xue et al. 2012a: 39.
		Anthocoptini	*Aculodes shiheziensis* Kuang, Lu & Zhang, 2005	Kuang et al. 2005: 81–82.
			*Aculops alopecuroides* Kuang, 1998	Kuang 1998: 410–411.
			*Aculops salixis* Xue, Song & Hong, 2007	Xue et al. 2012a: 41–42.
			*Aculus amygdali* Xue & Hong, 2005	Xue et al. 2012a: 43.
			*Aculus schlechtendali* (Nalepa, 1890)	Kuang 1995: 120–121.
			*Aculus tetanothrix* (Nalepa, 1889)	Kuang 1995: 131–132.
			*Tegolophus zizyphagus* (Keifer, 1939)	Kuang 1995: 146–147.
			*Tetra cuihuae* Xue, Song & Hong, 2006	Xue et al. 2012a: 54.
			*Tetra nitrariae* Li, Li, Zhang & Xue, 2014	Li et al. 2014: 348–351.
			*Tetra sativae* Li, Li, Zhang & Xue, 2014	Li et al. 2014: 339–343.
			*Tetra shiheziensis* Wang & Lu, 2004	Wang and Lu 2004: 266–267.
			*Tetra tianchiensis* Li, Li, Zhang & Xue, 2014	Li et al. 2014: 335–339.
			*Tetra tianschanicae* Li, Li, Zhang & Xue, 2014	Li et al. 2014: 330–334.
			*Tetra viciae* Li, Li, Zhang & Xue, 2014	Li et al. 2014: 343–348.
Diptilomiopidae	Rhyncaphytoptinae		*Rhyncaphytoptus fuyuniensis* sp. n.	This study
			*Rhyncaphytoptus yilisis* Song, Xue & Hong, 2007	Song et al. 2007: 63–65.
			*Rhyncaphytoptus ziziphi* Kuang, 2005	Kuang et al. 2005: 157–158.

## Materials and methods

Specimens of mites were collected from Xinjiang Uygur Autonomous Region, China. The morphological terminology used here follows Lindquist (1996). The generic classification of the eriophyoid mites is made according to Amrine et al. (2003), together with the comparison of genera erected after 2003. Specimens were cleared in Keifer’s booster and slides were mounted using modified Berlese medium (Amrine and Manson 1996). The number of measured specimens (n) is given within parentheses in the description. All specimens were examined, measured, taken photos and drawn with the aid of an Olympus Bx61 microscope using phase contrast. The measurements and drawings were based on the methods outlined by de Lillo et al. (2010) and abbreviations follow Amrine et al. (2003). For each species, the holotype female measurement precedes the corresponding range for paratypes (given in parentheses). For males, only ranges are given. All measurements are given in micrometres and are lengths unless specified. Type specimens are deposited at the Department of Plant Protection, College of Agriculture, Shihezi University, Xinjiang Uygur Autonomous Region, China.

## Results

### Family Eriophyidae Nalepa, 1898

#### Subfamily Cecidophyinae Keifer, 1966

##### Tribe Colomerini Newkirk & Keifer, 1975

###### Genus *Paracolomerus* Keifer, 1975

####### Paracolomerus gonglius sp. n.

Taxon classificationAnimaliaTrombidiformesEriophyidae

http://zoobank.org/36D41CF4-E10C-47F1-8635-3E2CC695A2CB

Fig. 2

######## Description.

FEMALE (n=6). Body vermiform, 187 (175–217, excluding gnathosoma), 50 (42–51) wide, 48 (40–47) thick; light yellow. **Gnathosoma** 24 (23–26), projecting obliquely down, pedipalp coxal setae (*ep*) 2 (2–3), dorsal pedipalp genual setae (*d*) 7 (6–8), unbranched, cheliceral stylets 21 (20–23). **Prodorsal shield** 30 (29–32), 37 (33–37) wide; median line almost complete, interrupted in the middle with short sloping lines on either side at the posterior end, admedian lines complete, submedian lines broken, with several short lines and granules on the lateral side; frontal shield lobe absent. Scapular tubercles near rear shield margin, 24 (23–24) apart, scapular setae (*sc*) 15 (14–15), projecting posterior. **Coxigenital region** with 5 (5–6) microtuberculated semiannuli. Coxal plates with several short lines, anterolateral setae on coxisternum I (*1b*) 7 (7–8), 11 (10–11) apart, proximal setae on coxisternum I (*1a*) 25 (25–28), 13 (12–13) apart, proximal setae on coxisternum II (*2a*) 44 (39–44), 24 (23–24) apart, tubercles *1b* and *1a* apart 5 (5–6), tubercles *1a* and *2a* 8 (7–8) apart. Internal coxisternal apodeme absent. Legs with usual series of setae. **Leg I** 27 (26–28), femur 8 (7–8), basiventral femoral setae (*bv*) 12 (12–14); genu 5 (4–5), antaxial genual setae (*l*'') 25 (23–26); tibia 6 (6–7), paraxial tibial setae (*l*') 7 (7–8), located in the middle; tarsus 7 (6–7), setae *ft*' 15 (14–15), setae *ft*'' 22 (20–22), seta *u*' 4 (4–5); tarsal empodium (*em*) 6 (5–6), simple, 5-rayed, tarsal solenidion (*ω*) 7 (7–8), rod-like. **Leg II** 25 (24–26), femur 7 (7–8), basiventral femoral setae (*bv*) 13 (13–14); genu 5 (4–5), antaxial genual setae (*l*'') 8 (8–10); tibia 5 (4–5); tarsus 7 (6–7), setae *ft*' 6 (6–7), setae *ft*'' 23 (23–25), seta *u*' 4 (4–5); tarsal empodium (*em*) 6 (5–6), simple, 5-rayed, tarsal solenidion (*ω*) 9 (9–10), rod-like. **Opisthosoma** dorsally arched, 64 (63–66) dorsal annuli, 63 (61–64) ventral annuli; microtubercles on the rear margin of the annuli, elliptical on the anterior part of dorsal annuli, linear and spiny on the posterior part of dorsal annuli and posterior part of ventral annuli, circular on the anterior part of ventral annuli. Setae *c2* 25 (25–27) on ventral annulus 9 (8–9), 47 (40–49) apart; setae *d* 65 (58–67) on ventral annulus 19 (18–21), 37 (34–37) apart; setae *e* 12 (12–14) on ventral annulus 32 (30–32), 20 (20–21) apart; setae *f* 29 (29–32) on 6th ventral annulus from rear, 21 (19–21) apart. Setae *h1* absent, *h2* 71 (69–74). **Genital coverflap** 11 (11–12), 22 (21–22) wide, coverflap with two rows of ridges, the basal one with 12 (11–13) longitudinal ridges, the other one with 9 (8–10) longitudinal ridges, proximal setae on coxisternum III (*3a*) 17 (17–20), 17 (17–18) apart.

MALE. Unknown.

**Figure 2.**
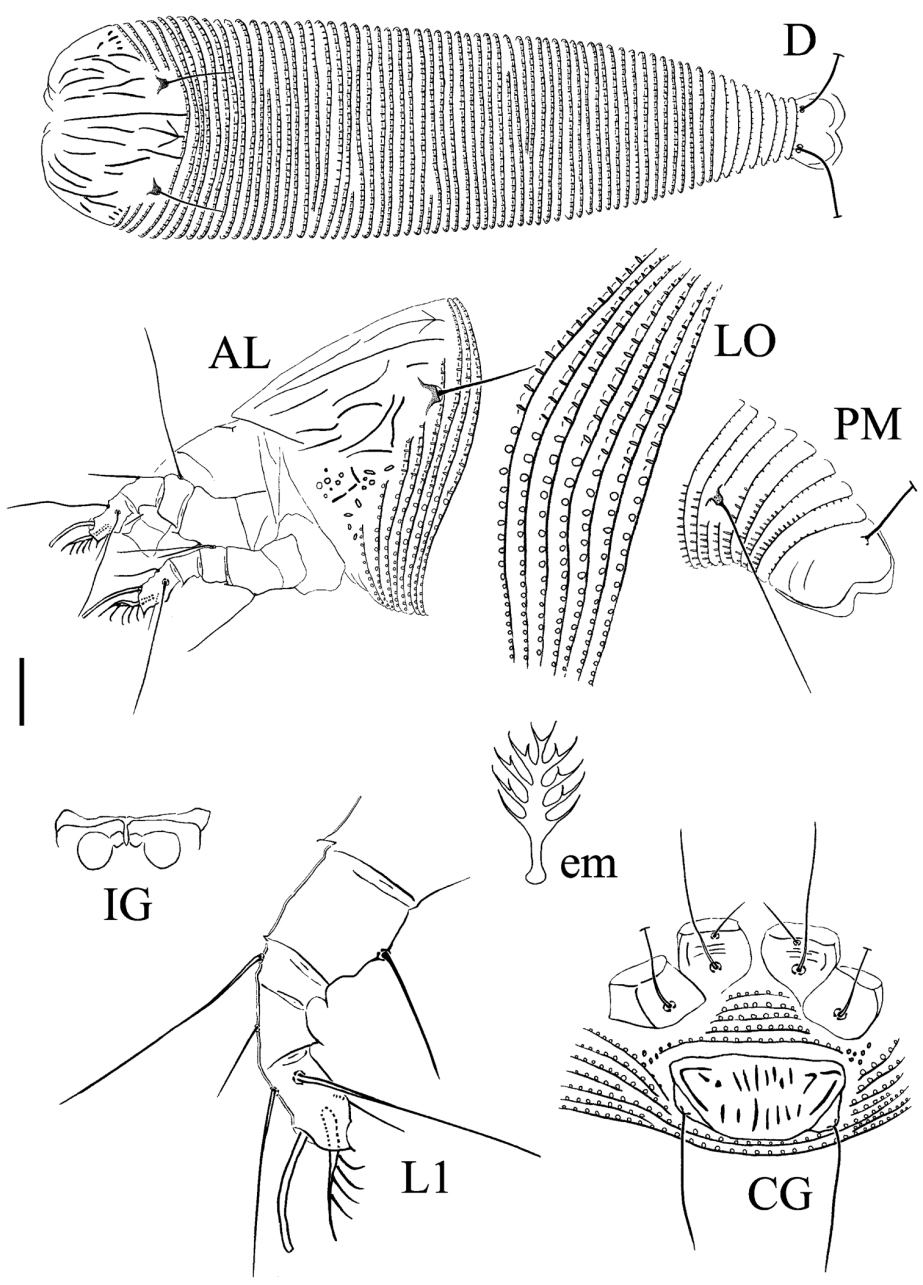
Schematic drawings of *Paracolomerus gonglius* sp. n.: **AL** Lateral view of anterior body region **CG** Female coxigenital region **D** Dorsal view **em** Empodium **IG** Internal female genitalia **LO** Lateral view of annuli **L1** Leg I **PM** Lateral view of posterior opisthosoma. Scale bar: 15 µm (**D**); 10 µm (**AL**, **CG**, **IG**, **PM**); 7.5 µm (**LO**); 5 µm (**L1**); 2.5 µm (**em**).

######## Type host plant.

*Rosa beggeriana* Schrenk ex Fisch. & C. A. Mey. (Rosaceae).

######## Relation to the host plant.

Vagrant on leaves; no apparent damage was observed.

######## Type locality.

Gongliu county, Xinjiang Uygur Autonomous Region, China (43°09'37"N, 81°36'34"E), elevation 1,396 m; 3 August 2014, coll. Ji-Wei Li.

######## Type material.

Holotype: female (slide number SHZU *Paracolomerus* 1.1, marked Holotype). Paratypes: 6 females mounted on 6 separate microscope slides.

######## Etymology.

The specific designation *gonglius* comes from the name of location, Gongliu, where the new species was collected.

######## Differential diagnosis.

All traits are in accordance with the type species *Paracolomerus casimiroae* Keifer, 1975 of the genus *Paracolomerus* (opisthosomal annuli subequal, legs with usual series of setae, scapular tubercles on rear shield margin, scapular setae projected posteriorly) except for ventral surface ornamentation of coxa I (lines do not circle around tubercles *1a* and meet at sternum in *Paracolomerus gonglius* sp. n.; lines originate at setae *1b*, circle distally around tubercles *1a* and meet at sternum, enclose most of the coxal surface in *Paracolomerus casimiroae*).

This species is similar to *Paracolomerus fopingacer* Xue, Song & Hong, 2011, from *Acer* sp. L. (Aceraceae), but can be differentiated from the latter by median line almost complete, with 5–6 short lines on the lateral sides of prodorsal shield (median line present for half, without short lines on the lateral sides in *Paracolomerus fopingacer*), frontal shield lobe absent (frontal shield lobe acuminate in *Paracolomerus fopingacer*) and 5-rayed empodium (6-rayed empodium in *Paracolomerus fopingacer*).

######## Remarks.

To date, only three species were reported from the genus *Paracolomerus*, *Paracolomerus casimiroae* Keifer, 1975, *Paracolomerus davidiae* Kuang & Hong, 1995 (in Kuang 1995) and *Paracolomerus fopingacer*.

#### Subfamily Phyllocoptinae Nalepa, 1892

##### Tribe Phyllocoptini Nalepa, 1892

###### Genus *Phyllocoptruta* Keifer, 1938

####### Phyllocoptruta beggerianae sp. n.

Taxon classificationAnimaliaTrombidiformesEriophyidae

http://zoobank.org/D33691AE-25DD-4A17-A854-E7C9925C96FF

Fig. 3

######## Description.

FEMALE (n=9). Body fusiform, 207 (182–207, excluding gnathosoma), 49 (46–51) wide, 43 (40–46) thick; white. **Gnathosoma** 28 (27–30), projecting obliquely down, pedipalp coxal setae (*ep*) 3 (2–3), dorsal pedipalp genual setae (*d*) 9 (8–9), unbranched, cheliceral stylets 26 (25–27). **Prodorsal shield** 42 (40–43), 43 (41–44) wide, median line formed by lined short lines, admedian lines complete and connected posteriorly, submedian lines present at the posterior half, with several short lines and granules; frontal shield lobe rounded, broad-based, 5 (4–5). Scapular tubercles ahead of rear shield margin, 23 (22–25) apart, scapular setae (*sc*) 16 (16–18), projecting forward and convergent. **Coxigenital region** with 9 (7–9) microtuberculated semiannuli. Coxal plates with several short lines and granules, anterolateral setae on coxisternum I (*1b*) 11 (10–11), 12 (11–12) apart, proximal setae on coxisternum I (*1a*) 27 (26–31), 10 (9–10) apart, proximal setae on coxisternum II (*2a*) 46 (42–46), 25 (23–25) apart, tubercles *1b* and *1a* apart 7 (6–7), tubercles *1a* and *2a* 9 (8–9) apart. Internal coxisternal apodeme 3 (3–4). Legs with usual series of setae. **Leg I** 36 (35–37), femur 10 (9–10), basiventral femoral setae (*bv*) 14 (13–15); genu 5 (4–5), antaxial genual setae (*l*') 22 (22–25); tibia 8 (8–9), paraxial tibial setae (*l*') 12 (10–12), located at 1/3 from dorsal base; tarsus 9 (8–9), setae *ft*' 19 (19–21), setae *ft*'' 22 (22–25), seta *u*' 10 (9–10); tarsal empodium (*em*) 8 (7–8), simple, 8-rayed, tarsal solenidion (*ω*) 10 (9–10), rod-like. **Leg II** 29 (28–30), femur 8 (8–9), basiventral femoral setae (*bv*) 16 (15–16); genu 4 (4–5), antaxial genual setae (*l*'') 8 (6–8); tibia 5 (5–6); tarsus 8 (7–8), setae *ft*' 9 (8–10), setae *ft*'' 23 (23–25), seta *u*' 9 (8–9); tarsal empodium (*em*) 8 (7–8), simple, 8-rayed, tarsal solenidion (*ω*) 10 (9–10), rod-like. **Opisthosoma** dorsally with a furrow in the middle; 35 (33–38) dorsal annuli, elliptical microtubercles on the rear margin; 74 (72–77) ventral annuli, microtubercles on the rear margin, circled on the anterior ventral annuli, and linear and spiny on the last posterior ventral annuli. Setae *c2* 28 (25–28) on ventral annulus 14 (12–14), 47 (45–48) apart; setae *d* 52 (48–52) on ventral annulus 28 (27–29), 35 (32–35) apart; setae *e* 33 (33–36) on ventral annulus 49 (47–51), 13 (12–14) apart; setae *f* 28 (26–29) on 6th ventral annulus from rear, 16 (16–17) apart. Setae *h1* 4 (3–4), *h2* 77 (75–83). **Genital coverflap** 13 (11–13), 19 (18–20) wide, coverflap with 3 transverse lines basally, 11 (11–13) longitudinal ridges distally, proximal setae on coxisternum III (*3a*) 43 (41–44), 15 (14–15) apart.

MALE (n=2). Similar in shape and prodorsal shield arrangement to female, 155–169. Prodorsal shield 32–35, 28–31 wide; scapular setae *sc* 16–17, 21–23 apart. Opisthosoma dorsally with a furrow, 32–37 annuli, ventrally with 74–81 annuli, dorsal and ventral microtubercles are similar to females. Male genitalia 17– 18 wide, setae *3a* 18– 20, 14–15 apart.

**Figure 3.**
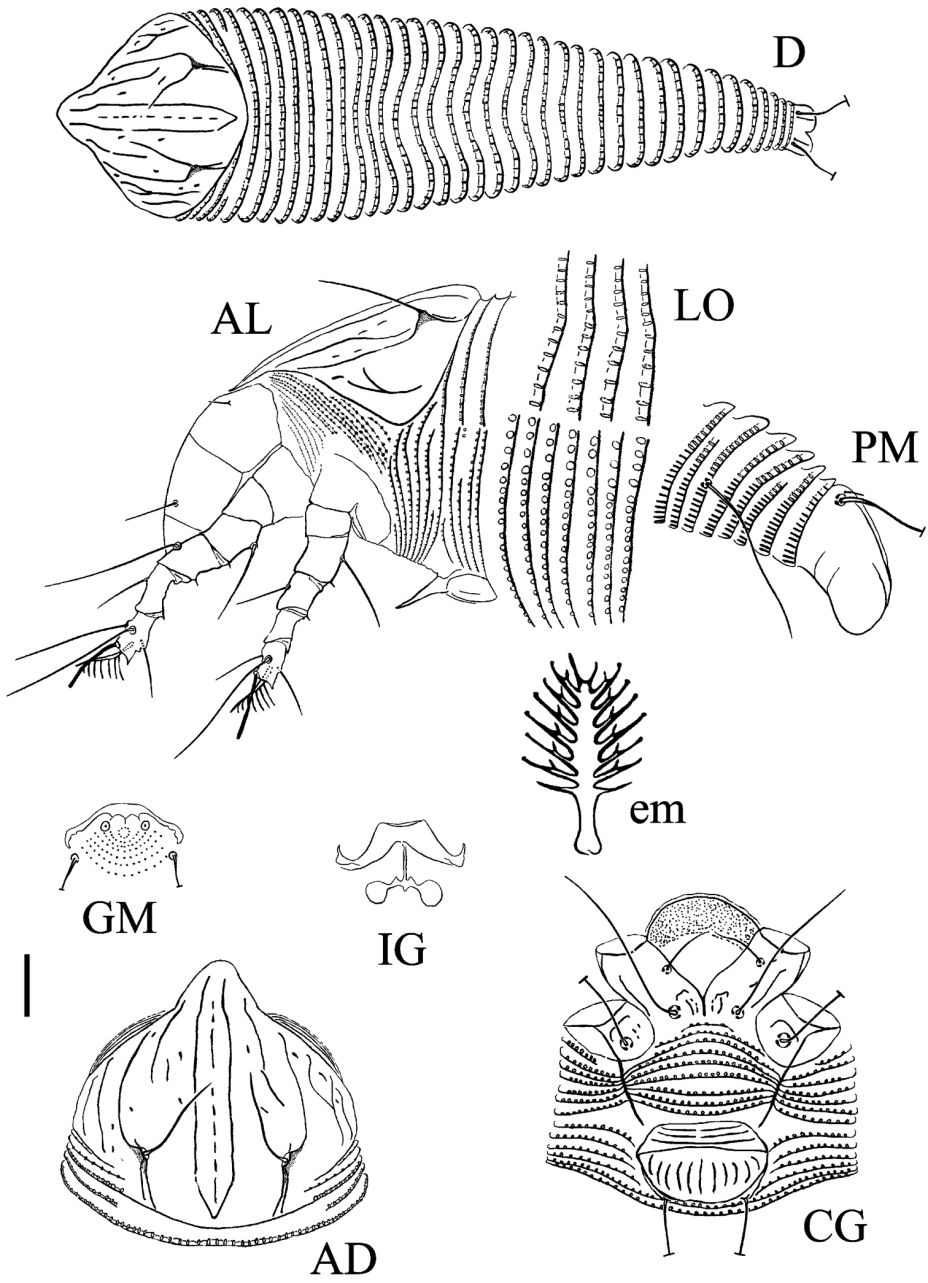
Schematic drawings of *Phyllocoptruta beggerianae* sp. n.: **AL** Lateral view of anterior body region **AD** Dorsal view of anterior body region **CG** Female coxigenital region **D** Dorsal view **em** Empodium **GM** Male genital region **IG** Internal female genitalia **LO** Lateral view of annuli **PM** Lateral view of posterior opisthosoma. Scale bar: 15 µm (**D**); 10 µm (**AD**, **AL**, **CG**, **IG**, **GM**, **PM**); 7.5 µm (**LO**); 2.5 µm (**em**).

######## Type host plant.

*Rosa beggeriana* Schrenk ex Fisch. & C. A. Mey. (Rosaceae).

######## Relation to the host plant.

Vagrant on leaves; no apparent damage was observed.

######## Type locality.

Xinyuan county, Xinjiang Uygur Autonomous Region, China (43°36'29"N, 82°17'56"E), elevation 758 m; 29 July 2014, coll. Ji-Wei Li.

######## Type material.

Holotype: female (slide number SHZU *Phyllocoptruta* 1.1, marked Holotype). Paratypes: 16 females and 2 males mounted on 18 separate microscope slides.

######## Etymology.

The specific designation *beggerianae* comes from the epithet of the host plant, *beggeriana*.

######## Differential diagnosis.

This species is similar to *Phyllocoptruta clematoclethra* Xue, Song & Hong, 2010, from *Clematoclethra* sp. Maxim. (Actinidiaceae), but can be differentiated from the latter by admedian lines connected posteriorly (admedian lines separate in *Phyllocoptruta clematoclethra*), scapular tubercles ahead of rear shield margin, scapular setae 16–18 (scapular tubercles on rear shield margin, scapular setae 3–4 in *Phyllocoptruta clematoclethra*), female genital coverflap with 3 transverse basal lines (coverflap without transverse lines in *Phyllocoptruta clematoclethra*) and 8-rayed empodium (5-rayed empodium in *Phyllocoptruta clematoclethra*).

### Family Diptilomiopidae Keifer, 1944

#### Subfamily Rhyncaphytoptinae Roivainen, 1953

##### Genus *Rhyncaphytoptus* Keifer, 1939

###### Rhyncaphytoptus fuyuniensis sp. n.

Taxon classificationAnimaliaTrombidiformesDiptilomiopidae

http://zoobank.org/CE644BB3-52C6-43E6-AAC7-06BC248177F7

Figs 4 , 5

####### Description.

FEMALE (n=8). Body fusiform, 256 (216–267, excluding gnathosoma), 60 (55–64) wide, 58 (54–62) thick; light yellow. Gnathosoma 61 (55–64), projecting downwards, pedipalp coxal setae (*ep*) 3 (2–3), dorsal pedipalp genual setae (*d*) 7 (6–7), unbranched, cheliceral stylets 83 (76–88). **Prodorsal shield** 29 (28–30) excluding the thin anterior process length from frontal lobe, 47 (46–49) wide, sub-triangular in anterior shape; long and flexible frontal lobe ending with a thin anterior process, the process extends for 14 (13–15). Median line very short, on 1/5 anterior part of prodorsal shield; admedian lines complete and connected at base with transverse lines, forming a vase-shaped outline; semicircled line between the scapular tubercles. Scapular tubercles ahead of rear shield margin, 30 (28–31) apart, scapular setae (*sc*) 47 (46–50), projecting forward. **Coxigenital region** with 15 (14–16) microtuberculated semiannuli. Coxal plates with 1–3 short lines, anterolateral setae on coxisternum I (*1b*) 10 (10–12), 10 (10–11) apart, proximal setae on coxisternum I (*1a*) 32 (29–33), 10 (10–11) apart, proximal setae on coxisternum II (*2a*) 45 (42–47), 29 (28–30) apart, tubercles *1b* and *1a* apart 7 (6–7), tubercles *1a* and *2a* 10 (9–11) apart. Internal coxisternal apodeme 7 (6–7). Legs with usual series of setae. **Leg I** 42 (41–43), femur 13 (12–14), basiventral femoral setae (*bv*) 13 (13–15); genu 7 (6–7), antaxial genual setae (*l*'') 23 (23–25); tibia 10 (10–11), paraxial tibial setae (*l*') 7 (7–8), located at 1/3 from dorsal base; tarsus 8 (7–8), setae *ft*' 20 (18–20), setae *ft*'' 26 (23–26), seta *u*' 5 (4–5); tarsal empodium (*em*) 8 (7–8), simple, 10-rayed, tarsal solenidion (*ω*) 8 (8–9), rod-like. **Leg II** 39 (38–40), femur 13 (12–13), basiventral femoral setae (*bv*) 14 (14–16); genu 6 (5–6), antaxial genual setae (*l*'') 9 (9–11); tibia 8 (7–9); tarsus 8 (8–9), setae *ft*' 10 (9–11), setae *ft*'' 30 (26–30), seta *u*' 5 (4–5); tarsal empodium (*em*) 8 (7–8), simple, 10-rayed, tarsal solenidion (*ω*) 10 (10–11), rod-like. **Opisthosoma** dorsally arched, 25 (20–25) dorsal annuli, 92 (90–104) microtuberculated ventral annuli; the anterior dorsal annuli smooth (for about 5/6 of them), the anterior ventral annuli with circled microtubercles (for about 2/3 of them), the posterior part of dorsal and ventral annuli with linear and spiny microtubercles. Setae *c2* 13 (12–14) on ventral annulus 19 (17–21), 59 (53–61) apart; setae *d* 51 (46–51) on ventral annulus 37 (36–42), 45 (41–45) apart; setae *e* 26 (26–29) on ventral annulus 55 (53–64), 25 (24–26) apart; setae *f* 30 (27–30) on 7th ventral annulus from rear, 21 (20–22) apart. Setae *h1* 3 (3–4), *h2* 75 (70–79). **Genital coverflap** 15 (14–16), 30 (28–30) wide, coverflap with many granules basally, proximal setae on coxisternum III (*3a*) 14 (13–14), 20 (19–21) apart.

MALE (n=5). Similar in shape and prodorsal shield arrangement to female, 202–243. Prodorsal shield 22–25 without the frontal lobe length, 45–48 wide; scapular setae *sc* 40–46, 27–30 apart. Opisthosoma dorsally with 19–21 annuli; ventrally with 78–85 annuli, dorsal and ventral microtubercles are similar to females. Male genitalia 21–22 wide, setae *3a* 12–13, 18–20 apart.

**Figure 4.**
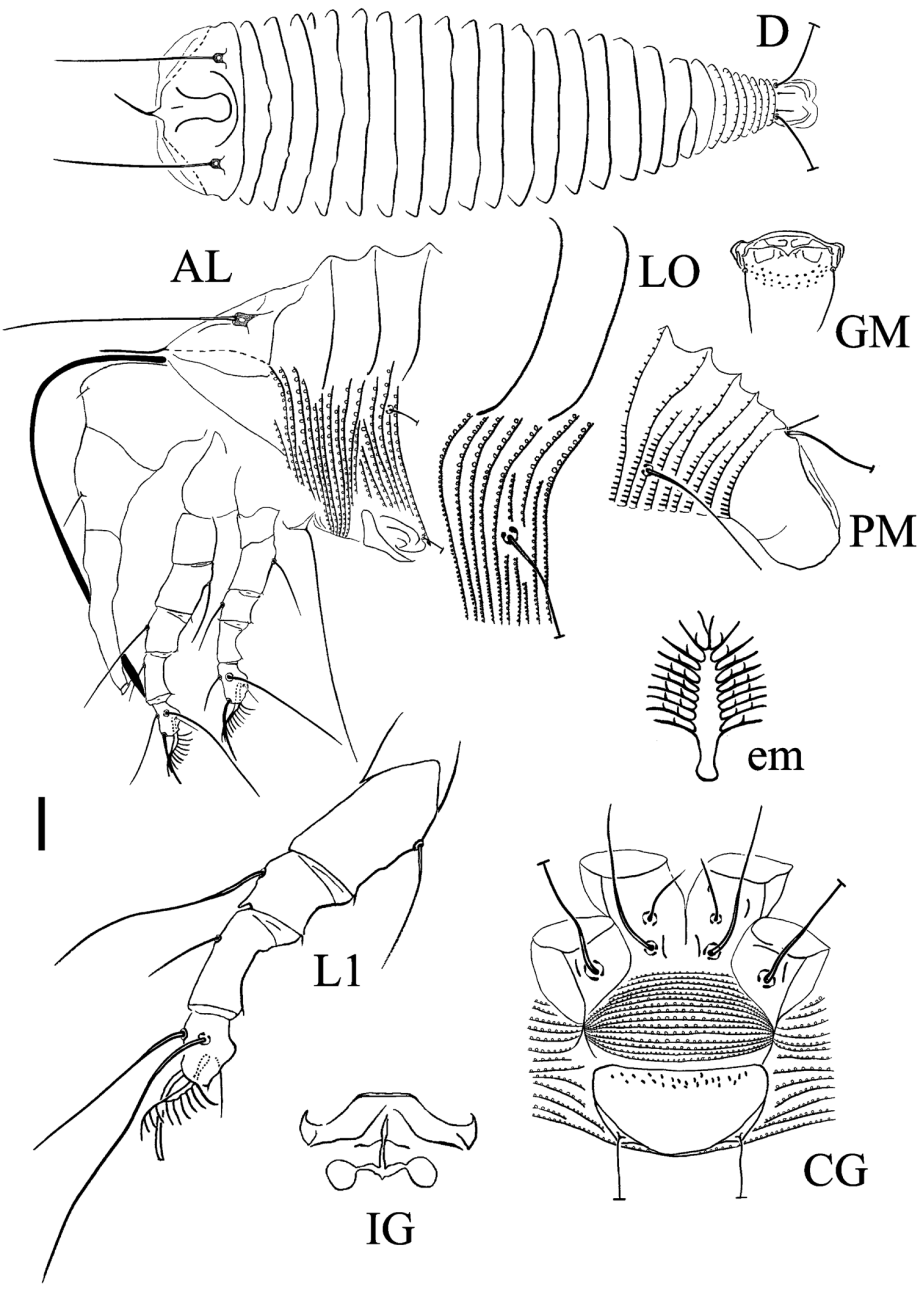
Schematic drawings of *Rhyncaphytoptus fuyuniensis* sp. n.: **AL** Lateral view of anterior body region **CG** Female coxigenital region **D** Dorsal view **em** Empodium **GM** Male genital region **IG** Internal female genitalia **LO** Lateral view of annuli and setae *d* **L1** Leg I **PM** Lateral view of posterior opisthosoma. Scale bar: 15 µm (**D)**; 10 µm (**AL**, **CG**, **IG**, **GM**, **PM**); 7.5 µm (**LO**); 5 µm (**L1**); 2.5 µm (**em**).

**Figure 5.**
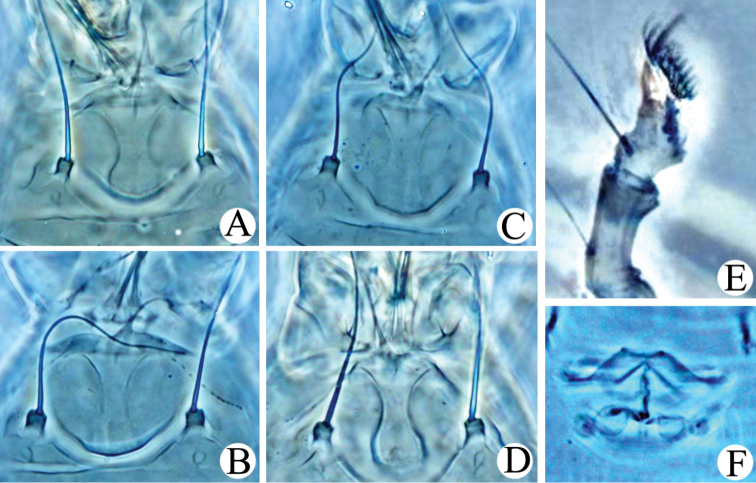
*Rhyncaphytoptus fuyuniensis* sp. n.: **A**–**D** Frontal lobe **E** Tibia and tarsus of Leg I **F** Female internal genitalia.

####### Type host plant.

*Cotoneaster ignavus* E. L. Wolf (Rosaceae).

####### Relation to the host plant.

Vagrant on leaves; no apparent damage was observed.

####### Type locality.

Fuyun county, Xinjiang Uygur Autonomous Region, China (47°17'39"N, 89°58'26"E), elevation 1,359 m; 15 August 2014, coll. Ji-Wei Li.

####### Type material.

Holotype: female (slide number SHZU *Rhyncaphytoptus* 7.1, marked Holotype). Paratypes: 12 females and 15 males mounted on 27 separate microscope slides.

####### Etymology.

The specific designation *fuyuniensis* comes from the name of location, Fuyun, where the new species was collected.

####### Differential diagnosis.

This species is similar to *Rhyncaphytoptus buxifoliae* Song, Xue & Hong, 2009, from *Cotoneaster buxifolius* Lindl. (Rosaceae), but can be differentiated from the latter by median line very short, on 1/5 anterior part of prodorsal shield (prodorsal shield with incomplete median line on posterior 1/2 in *Rhyncaphytoptus buxifoliae*), scapular tubercles small (scapular tubercles 5–13 long in *Rhyncaphytoptus buxifoliae*) and with a long and flexible frontal lobe (lack a distinct, long frontal lobe in *Rhyncaphytoptus buxifoliae*).

## Supplementary Material

XML Treatment for Paracolomerus gonglius

XML Treatment for Phyllocoptruta beggerianae

XML Treatment for Rhyncaphytoptus fuyuniensis
